# Predictors for late genitourinary toxicity in men receiving radiotherapy for high-risk prostate cancer using planned and accumulated dose

**DOI:** 10.1016/j.phro.2023.100421

**Published:** 2023-02-02

**Authors:** Ashley Li Kuan Ong, Kellie Knight, Vanessa Panettieri, Mathew Dimmock, Jeffrey Kit Loong Tuan, Hong Qi Tan, Caroline Wright

**Affiliations:** a Division of Radiation Oncology, National Cancer Centre Singapore, Singapore; b Department of Medical Imaging and Radiation Sciences, Monash University, Clayton, VIC, Australia; c Central Clinical School, Monash University, Melbourne, VIC, Australia; d Department of Physical Sciences, Peter MacCallum Cancer Centre, Melbourne, VIC, Australia; e School of Allied Health Professions, Keele University, Staffordshire, UK

**Keywords:** Accumulated dose, Multivariate model, Genitourinary toxicity, Volumetric image-guidance, High-risk prostate cancer

## Abstract

**Background and purpose:**

Significant deviations between bladder dose planned (D_P_) and dose accumulated (D_A_) have been reported in patients receiving radiotherapy for prostate cancer. This study aimed to construct multivariate analysis (MVA) models to predict the risk of late genitourinary (GU) toxicity with clinical and D_P_ or D_A_ as dose-volume (DV) variables.

**Materials and methods:**

Bladder D_A_ obtained from 150 patients were compared with D_P_. MVA models were built from significant clinical and DV variables (p < 0.05) at univariate analysis. Previously developed dose-based-region-of-interest (DB-ROI) metrics using expanded ring structures from the prostate were included. Goodness-of-fit test and calibration plots were generated to determine model performance. Internal validation was accomplished using Bootstrapping.

**Results:**

Intermediate-high D_A_ (V_30-65 Gy_ and DB-ROI-20–50 mm) for bladder increased compared to D_P_. However, at the very high dose region, D_A_ (D_0.003 cc_, V_75 Gy,_ and DB-ROI-5–10 mm) were significantly lower. In MVA, single variable models were generated with odds ratio (OR) < 1. DB-ROI-50 mm was predictive of Grade ≥ 1 GU toxicity for D_A_ and D_P_ (D_A_ and D_P_; OR: 0.96, p: 0.04) and achieved an area under the receiver operating curve (AUC) of > 0.6. Prostate volume (OR: 0.87, p: 0.01) was significant in predicting Grade 2 GU toxicity with a high AUC of 0.81.

**Conclusions:**

Higher D_A_ (V_30-65 Gy_) received by the bladder were not translated to higher late GU toxicity. DB-ROIs demonstrated higher predictive power than standard DV metrics in associating Grade ≥ 1 toxicity. Smaller prostate volumes have a minor protective effect on late Grade 2 GU toxicity.

## Highlights

• Accumulated Bladder dose was significantly higher at the intermediate-high dose region (V30-65 Gy) compared to bladder planned dose.
• Accumulated bladder dose was significantly lower at the very high dose region (V70-75 Gy) compared to the planned bladder dose.
• Dose-based region of interest structures were better predictors than dose-volume metrics for the bladder.
• Higher accumulated bladder dose was not translated to an increased in GU toxicity compared to planned dose.
• Smaller prostate volumes have a minor protective effect on Grade 2 late GU toxicity.

## 1 Introduction

High-risk prostate cancer (HR-PCa) accounts for about 15 % of all prostate cancer diagnosis with a higher likelihood of metastatic relapse after definitive treatment [1], [2]. The application of modern radiotherapy (RT) technologies indicates that dose escalation and hypofractionated regimens have the potential to improve biochemical disease-free survival in HR-PCa [3], [4]. Often, the bladder constraints can be achieved on the dose-volume histogram (DVH) metrics using the planning CT (pCT) acquired when the patient follows a bladder filling protocol to displace the bowels from the high dose region [5]. However, these treatment plans generated on static pCT, do not account for volumetric variations of the bladder during treatment [6]. Reported genitourinary (GU) toxicity remains high, especially in patients with HR-PCa which requires prophylactic pelvic lymph nodes (PLNs) irradiation as the bladder is positioned between the PLNs [7].

Inter-fractional volumetric changes during the course of RT could affect the actual dose received by the target volumes and surrounding organs at risk (OARs) [8], [9], [10]. To date, most of the reported studies involve the prostate only as the target volume and use the dose planned (D_P_) as dose-volume (DV) variables for associations with GU toxicity [11], [12]. There is limited work being performed using dose accumulated (D_A_) as DV variables in the setting of HR-PCa with PLNs irradiation [12], [13]. Additionally, the cohort size in these published works were small. The lack of robust data in this aspect could be due to the time-consuming and resource-intensive nature of constructing and streamlining a dose accumulation workflow [14], [15]. The developed and validated dose accumulation workflow from our previous work was used to generate the accumulated dose (D_A_) for the bladder, employing the patient’s daily CBCT images [16]. Apart from the DV component, GU toxicity has been reported to be influenced by clinical variables such as patient-related factors, medications, and the occurrence of acute GU toxicity within three months from RT [17], [18], [19], [20].

In this study, we hypothesise that the multivariate analysis (MVA) models generated using D_A_ for the DV component are more predictive than D_P_ in determining the occurrence of late Grade ≥ 1 and Grade 2 GU toxicity in HR-PCa. None of the patients in this study experienced Grade 3 and 4 GU toxicity. The goals of this study were firstly to evaluate the DV differences between D_A_ and D_P_ for the bladder. Secondly, MVA models with the toxicity endpoints of developing late Grade ≥ 1 and 2 GU toxicity were independently assessed using either D_A_ or D_P_ and the clinical variables.

## 2 Materials and methods

In this study, a total of 150 HR-PCa patients with prophylactic PLNs irradiation treated in our institution from January 2016 to December 2019 were retrospectively recruited. The median follow-up (FU) for the entire cohort was 57 months, ranging from 31.8 to 77.0 months. Ethics approval was obtained from the centralized institutional review board (CIRB ref: 2019/2018). Table 1 presented patients’ clinical variables, acute and late toxicity profiles. Similar methodology has been adopted from previous publication based on clinical and DV associations with late gastrointestinal (GI) toxicity [21].

**Table 1:** Patient’s clinical variables, acute and late toxicity profiles. The numbers in brackets are percentages rounded down to the nearest integer.

**Clinical variables**	**N = 150 cases**
Age at diagnosis, yrs.; mean [±SD]	71 [6]
BMI, kg/m^2^; mean [±SD]	25 [3.8]
Gleason score; mean [±SD]	8 [1]
≤ 7 (%); > 7 (%); Not known (%)	62 (42); 86 (57); 2 (1)
cT-stage (AJCC 8th edition)	
≤ 2b; > 2b (%)	75 (50); 75 (50)
Baseline PSA (ng/mL); mean [±SD]	35 [48]
**Medications**	
Anti-hypertensive (%) No; Yes	76 (51); 74 (49)
Metformin (%) No; Yes	117 (78); 33 (22)
Statins (%) No; Yes	98 (65); 52 (35)
TURP (%) No; Yes; Not known	136 (90); 13 (9); 1 (1)
ADT (%) ≤ 6 months; > 6 months	39 (26); 111 (74)
RT prescription (%) ≤ 74 Gy; > 74 Gy	88 (59); 62 (41)
**Organ volumes**	
Prostate vol. (cm^3^); mean [±SD]	36.8 [19]
Bladder vol. (cm^3^); mean [±SD]	209 [88.9]
**Overall acute toxicity (%)**	
Grade 0–1; Grade 2	118 (79); 37 (25)
**Late toxicity (%)**	
Urinary frequency Grade 1; Grade 2	6 (4); 3 (2)
Urinary urgency Grade 1; Grade 2	8 (5); 1 (1)
Urinary incontinence Grade 1; Grade 2	15 (10); 2 (1)
Cystitis Grade 1; Grade 2	12 (8); 5 (3)
**Overall late toxicity (%)**	
Grade 0–1; Grade 2	41 (27); 11 (7)

Abbreviations: BMI = body mass index, AJCC = American Joint Committee on Cancer antigen, PSA = prostate specific antigen; TURP = transurethral resection of the prostate; ADT = androgen deprivation therapy; GU = genitourinary; SD = standard deviation.

### 2.1 CT-Simulation and treatment planning

Patients were simulated in a supine position with arms on their chest using a leg immobilizer. Before CT-simulation and each RT session, patients were advised to adhere to the bladder filling protocol (2–3 cups; 400–600 ml of water, 30-60mins) and were encouraged to empty their bowels. CT-simulation was undertaken with 2.5 mm slice thickness (120kVp, GE LightSpeed RT 16). Clinical target volumes (CTVs) were defined as the prostate, seminal vesicles (SVs), PLNs with superior extend at L5/S1 interspace for phase 1 (Ph1), and a coned down CTV for phase 2 (Ph2) defined as the prostate and proximal 1 cm of the SVs. Planning target volumes (PTVs) comprised of an anisotropic expansion margins of 5 mm posteriorly and 5–8 mm to all other directions from the CTVs. Dose prescriptions comprising of 46–54 Gy (23–27 fractions) and an additional 24–28 Gy (12–14 fractions) were prescribed to Ph1 and Ph2 respectively. Both phases were planned using 10 MV energy, dual arc Volumetric Modulated Arc Therapy (VMAT) technique.

### 2.2 Dose based-region of interest (DB-ROI)

DB-ROI structures were created using an automated process utilizing a customized workflow in MIM (MIMVista® v6.9, MIM Software Inc., Cleveland OH USA) [22] (refer to supplemental Fig. S1). The mean dose derived from the novel DB-ROI method was used as DV variables together with the standard DV values in MVA. This method accounted for the volumetric changes of the bladder at a fixed distance from the prostate surface, thereby minimizing the uncertainties in defining the bladder trigone where correlation with GU toxicity has been reported [23].

### 2.3 Dose accumulation workflow

A customized dose accumulation workflow that was able to accommodate two sequential treatment phases using MIM was developed. Details of the workflow building and validations of the deformable image registration (DIR) algorithm have been previously described [22]. The generation of D_A_ was based on patients’ daily CBCT scans acquired as part of their target localization procedure before treatment delivery. The scans were acquired in a half-fan mode (45 cm field-of-view, 120 kVp) scan, and reconstructed to 2.5 mm slice thickness (Varian on-board imaging v2.1, Varian Medical Systems, Palo Alto, CA).

### 2.4 GU toxicity assessment and documentation

Late GU toxicity was recorded after three months post-RT, six-monthly for five years followed by yearly thereafter. In this study, the incidence of maximum toxicity grading for Grade ≥ 1 and 2 GU toxicity defined at two years post-RT FU were used as the examined clinical toxicity endpoints. Grade ≥ 1 and Grade 2 toxicity are defined as patients having mild-severe (Grade 1–2) and severe (Grade 2 only) late GU toxicity respectively. Previously toxicity records graded using the Radiation Therapy Oncology Group (RTOG) criteria were reviewed and re-graded by the National Cancer Institute Common Terminology Criteria for Adverse Events (version 4.03; CTCAE) by the radiation oncologist from the study team. The GU toxicities were defined as urinary frequency, urinary urgency, urinary incontinence, and cystitis.

### 2.5 Statistical analysis and modelling

The primary clinical outcome of this study was the occurrence of Grade ≥ 1 and 2 GU toxicity measured at two years post-RT FU. Descriptive statistics (e.g., means ± standard deviation, medians with interquartile ranges) were calculated. For the DV analysis comparing D_P_ and D_A_ values, a parametric two-sided *t*-test was used after performing a normality test using the Shapiro-Wilk test and evaluated visually with QQ-plots and histograms. A p-value of < 0.05 was deemed significant. Highly correlated variables tested using Pearson correlation test (r ≥ 0.8) were removed. Univariate logistic analysis (UVA) was performed on individual clinical and DV variables to define associations with late clinical endpoints. For the DV variables, D_A_ and D_P_ were being analysed separately with the defined GU toxicity. Variables with p-values of < 0.05 were statistically significant [24], [25]. Significant variables at the UVA level were used for the subsequent MVA using an enter/remove method to identify the independent predictors for the final MVA model, whereby p < 0.05 was considered statistically significant [26]. Results were reported as odds ratios (OR), 95 % confidence intervals (CIs), and p-values.

Model performance was measured for its calibration results and discriminative ability. For model calibration, the Hosmer-Lemeshow p-value (p-HL) goodness of fit test was used to generate the calibration plot. The observed outcomes were divided into quartiles to obtain the observed probabilities and were plotted against the predicted probabilities for binary dependent variables [20]. The ability of the models to distinguish patients with the defined clinical outcomes was evaluated using the area under the receiver operating characteristic curve (AUC). An ideal correlation corresponds to an AUC of 1. An AUC of ≥ 0.6 and minimum 95 % CI ≥ 0.5 was considered statistically significant [27]. Internal validation was accomplished using bootstrapping, in which resampling with replacement techniques was performed 1000 times on the original dataset and recalculated during the variable selection process adjusting for model optimism [19], [28]. Best fit predictors with 95 % CI were obtained. All analyses were performed using SPSS statistics (IBM Corp. v27.0. Armonk, NY) and R software (https://www.r-project.org/, version 4.0, Vienna, Austria).

## 3 Results

### 3.1 Dose-volume analysis between D_A_ and D_P_ for the bladder

For the bladder, ${\overset{¯}{D}}_{Dmean\;Gy\;}^{blad}$ and ${\overset{¯}{D}}_{V30-65\;Gy}^{blad}$ for D_A_ was significantly higher than D_P_ except at the very high dose region (${\overset{¯}{D}}_{D0.03\;Gy}^{blad}$ and ${\overset{¯}{D}}_{V75\;Gy}^{blad}$) whereby the dose calculated for D_P_ was significantly higher (p < 0.001) (refer to Table 2). On average, a dose difference of>4 Gy for D_A_ could be seen at the intermediate dose region (${\overset{¯}{D}}_{\;V35-45Gy}^{blad}$).

**Table 2:** Evaluation of planned and accumulated bladder dose based on dose-volume metrics. Mean dose difference between the accumulated and planned were presented.

Parameters	${\overset{¯}{D}}_{P}$(±SD), Gy/%	${\overset{¯}{D}}_{A}$(±SD), Gy/%	${\overset{¯}{D}}_{A}$- ${\overset{¯}{D}}_{P}$(±SD), Gy/%	p - value
Dmean [Gy]	47.3 (6.1)	48.7 (6.6)	1.4 (2.1)	p < 0.001
D0.03 cc [Gy]	79.2 (2.1)	78.1 (2.0)	−1.1 (0.4)	p < 0.001
V30 Gy [%]	83.6 (14.8)	87.1 (14.0)	3.5 (4.2)	p < 0.001
V35 Gy [%]	71.8 (15.5)	76.2 (16.1)	4.4 (5.2)	p < 0.001
V40 Gy [%]	59.2 (14.8)	64.0 (16.8)	4.8 (5.6)	p < 0.001
V45 Gy [%]	48.8 (14.3)	52.7 (16.9)	4.0 (5.8)	p < 0.001
V50 Gy [%]	39.1 (14.3)	42.6 (16.7)	3.5 (5.6)	p < 0.001
V55 Gy [%]	31.7 (13.5)	34.5 (15.8)	2.8 (5.2)	p < 0.001
V60 Gy [%]	26.0 (12.3)	27.9 (14.3)	2.0 (4.7)	p < 0.001
V65 Gy [%]	20.9 (10.7)	21.9 (12.2)	1.0 (4.1)	p < 0.001
V70 Gy [%]	16.3 (8.8)	16.1 (9.7)	−0.2 (3.5)	p = 0.48
V75 Gy [%]	10.6 (6.2)	8.6 (6.4)	−2.0 (2.9)	p < 0.001

Abbreviations: ${\overset{¯}{D}}_{A}$= mean dose accumulated, ${\overset{¯}{D}}_{P}$= mean dose planned; SD = standard deviation; D_mean_ = mean dose; Dx [Gy] = dose [Gy] received by the specified × volume (%); Vx [%] = volume of the organ [%] receiving the specified × dose (Gy).

### 3.2 Dose-based ROI analysis between D_A_ and D_P_ for the bladder

The absolute mean DV values for bladder ${\overset{¯}{D}}_{A}$, ${\overset{¯}{D}}_{P}$ and the difference in dose (${\overset{¯}{D}}_{A}$ - ${\overset{¯}{D}}_{P}$), Gy were calculated for all patients per ROI. ${\overset{¯}{D}}_{ROI\;5-50\;mm}^{blad}$ were shown in Table 3. For ${\overset{¯}{D}}_{A\;ROI\;20-50\;mm}^{blad}$ , the obtained dose difference was significantly higher as compared to D_P_ (${\overset{¯}{D}}_{A}$ - ${\overset{¯}{D}}_{P}$, p < 0.001). The greatest dose difference of 2.9 ± 3.4 Gy was observed at ${\overset{¯}{D}}_{A\;ROI\;25\;mm}^{blad}$. At ${\overset{¯}{D}}_{ROI\;5-10\;mm}^{blad}$ region, D_A_ on average was significantly lower compared to D_P_ (p < 0.001).

**Table 3:** Evaluation of accumulated and planned dose delivered to bladder ROIs. Mean dose difference between the accumulated and planned were presented.

Bladder (mm)	${\overset{¯}{D}}_{P}$(±SD), Gy	${\overset{¯}{D}}_{A}$(±SD), Gy	${\overset{¯}{D}}_{A}$- ${\overset{¯}{D}}_{P}$(±SD), Gy	p-value
5	77.5 (1.9)	77.0 (2.2)	−0.5 (1.0)	p < 0.001
10	76.4 (2.5)	74.8 (3.2)	−1.6 (1.8)	p < 0.001
15	69.5 (4.5)	69.1 (4.8)	−0.4 (2.9)	p = 0.13
20	58.8 (5.4)	61.1 (5.7)	2.3 (3.4)	p < 0.001
25	50.7 (5.6)	53.5 (5.9)	2.9 (3.4)	p < 0.001
30	45.2 (5.8)	47.6 (6.0)	2.4 (3.0)	p < 0.001
35	41.4 (5.9)	43.2 (6.0)	1.8 (2.6)	p < 0.001
40	38.0 (6.6)	39.4 (6.7)	1.4 (2.2)	p < 0.001
45	35.7 (6.9)	36.5 (7.4)	1.1 (2.0)	p < 0.001
50	32.5 (9.2)	33.4 (9.2)	0.9 (1.8)	p < 0.001

Abbreviations: ROI = region of interest; ${\overset{¯}{D}}_{A}$ = mean dose accumulated, ${\overset{¯}{D}}_{P}$= mean dose planned; SD = standard deviation.

### 3.3 MVA modelling and model performance evaluation

**Fig. 1:**
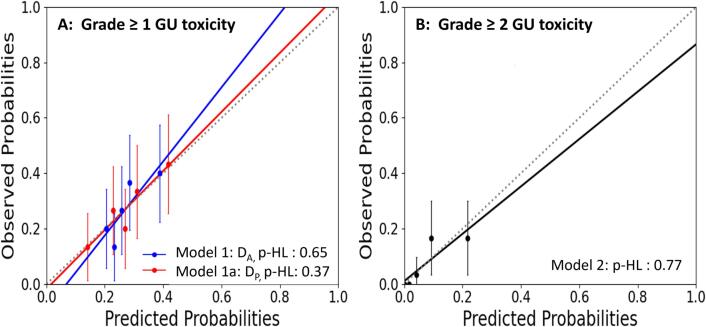
Calibration plots (predicted vs observed probabilities) for Grade ≥ 1 (A) and Grade 2 GU toxicity (B). The 45° dotted line represents the reference line where y = x.

For MVA modelling, clinical variables were evaluated separately with the DV variables D_A_ and D_P_ respectively based on the significant predictors found in UVA (refer to supplemental Table S1). Three statistically significant single variable models (p < 0.05) were achieved, correlating to the development of late Grade ≥ 1 and 2 GU toxicity (see Table 4). All the obtained models have an OR < 1 for the defined clinical endpoints. The models demonstrated good performance by attaining an AUC of ≥ 0.6, with Model 2 having the highest AUC of 0.81 (see Table 5). Similarly, the models were also well-calibrated whereby the obtained p-HL values were ≥ 0.05, indicating that the predicted probability is comparable to the actual observed events as demonstrated in Fig. 1.

**Table 4:** Resultant single variable models generated from MVA. Odds ratio with 95% confidence interval were presented.

MVA Models	Clinical and D_A_ or D_P_	Variables	OR	95 % CI	p-value
Model 1	D_A_, Grade ≥ 1	${\overset{¯}{D}}_{A\;ROI\;50\;mm\;(Gy)}^{\mathrm{blad}}$	0.96	0.93 – 0.99	P = 0.04
Model 1a	D_P_, Grade ≥ 1	${\overset{¯}{D}}_{P\;ROI\;50\;mm\;(Gy)}^{\mathrm{blad}}$	0.96	0.93 – 0.99	P = 0.04
Model 2	Grade 2	Prostate volume	0.87	0.79–0.96	P = 0.01

Abbreviations: D_A_ = dose accumulated; DP = dose planned; ROI = region of interest; OR = odds ratio;

**Table 5:** Mean area under the ROC curve (AUC) obtained for each model to evaluate model performance of the MVA models.

MVA Models	Model performance							
	Clinical vs D_A_ or D_P_	p-HL	R^2^	AUC	p-value	95 % CI	Sensitivity	1-Specificity
Model 1	D_A_, Grade 1	0.65	0.04	0.63	p < 0.01	0.53–0.73	0.71	0.45
Model 1a	D_P_, Grade 1	0.37	0.04	0.62	p < 0.05	0.52–0.73	0.71	0.46
Model 2	Grade 2	0.77	0.23	0.81	p < 0.001	0.72–0.90	0.91	0.32

Abbreviations: MVA = multivariate, D_A_ = dose accumulated, D_P_ = dose planned, GI = gastrointestinal; p-HL = Hosmer-Lemeshow p-values for goodness of fit test; AUC = area under the receiver operator curve; R^2^ = Pseudo R^2^.

### 3.4 MVA regression analysis for Grade ≥ 1 and 2 GU toxicity at 2 years post-RT FU

For Grade ≥ 1 GU toxicity, ${\overset{¯}{D}}_{ROI\;50\;mm}^{blad}$ was the only significant DV predictor for Model 1 and 1a with D_A_ and D_P_ respectively (p < 0.05), although D_A_ achieved a slightly higher mean dose of 0.9 Gy compared to D_P_ (refer to Table 4). An OR of < 1 was obtained for Model 1 and 1a, indicating that there was a marginal prophylactic effect for low dose on the risk of Grade ≥ 1 GU toxicity. For Grade 2 GU toxicity in Model 2, every 1 cm^3^ increment in prostate volume has a corresponding 13 % reduction in toxicity event. None of the DV predictors were significant in defining this clinical outcome.

## 4 Discussions

This study hypothesised that MVA models obtained using D_A_ as the DV component are more predictive than D_P_ in associating with late GU toxicity. This is one of the largest studies to date using VMAT technique and with D_A_ obtained from a customized automated workflow to account for patient’s inter-fractional organ motion, in addition to patient’s clinical variables for model construction.

For Models 1 and 1a, the risk of developing Grade ≥ 1 late GU toxicity was reduced moderately with the corresponding increase in dose received by the ${\overset{¯}{D}}_{ROI\;50\;mm}^{blad}$ (low-intermediate range) for both D_A_ and D_P_ [29]. This could be due to a prophylactic effect of low dose on GU toxicity. However, Marcello et al. [30] conducted a study on 1071 men treated using 3D RT found that low-intermediate doses to the extraprostatic urethra were associated with the risk of developing late GU toxicity. The findings contrasted with our findings as urethra was not contoured and assessed in this study and moreover, the occurrence of late GU toxicity has been reported to be beyond 2 years [31]. In the DV analysis phase, D_A_ for the bladder was significantly higher for most of the dose range for ${\overset{¯}{D}}_{V30-65\;Gy}^{blad}$ and ${\overset{¯}{D}}_{ROI\;20-50\;mm}^{blad}$. This could be due to an overall reduction in bladder volume throughout RT, thus resulting in a larger volume of the bladder being bathed in the PLNs dose range [13]. This observation validated the results reported in our recently published work demonstrating the correlations between dose received by OARs and the volumetric changes using patients’ CBCTs on 20 HR-PCa patients [16], [22]. Despite having a bladder filling protocol in place, maintaining bladder consistency during RT is challenging due to factors such as patient hydration status, co-morbidities (e.g., diabetes), and the intake of diuretics [5]. As compared to the bladder D_P_, higher values for bladder D_A_ obtained for BD-ROI metrics do not contribute to an enhanced risk of late Grade ≥ 1 GU toxicity in this study. These findings are corroborated by studies involving full bladder protocols in conventionally fractionated RT for PCa [6], [32].

For Model 2, none of the DV variables were significant in predicting late Grade 2 GU toxicity. This finding might be due to the low toxicity rates (n = 11) in this study contributed by the use of inverse planning technique with optimal OARs sparing [33] and the high tolerability of the bladder tissues to radiation [34]. Moreover, other co-factors such as patient’s genomic and proteomic features might play an important role in the risk of developing GU toxicity [35]. Prostate volume was the only significant clinical predictor that has shown to have a minor protective effect on the risk of developing late Grade 2 GU toxicity. Studies investigating the impact of pre-treatment prostate volumes on GU toxicity found that larger prostate volumes (median > 50 cm^3^) correlate to higher rates of acute GU toxicity, but symptoms were resolved within a year [36], [37]. In this study, the smaller prostate volumes observed (mean: 36.8 cm^3^, SD: ± 19 cm^3^) could be due to the routine use of neoadjuvant ADT as the standard of care for patients with HR-PCa [38], [39]. This is in parallel to studies reporting that a corresponding reduction in Grade ≥ 2 GU toxicity has been observed in patients with neoadjuvant RT as compared to patients treated with RT alone [40], [41].

There were several limitations to this study. Firstly, the toxicity outcomes were physician-reported rather than patient-reported (PROs). The rates of underestimation of the actual Grade ≥ 1 and Grade 2 GU toxicity may be present as there is a low agreement between physician and patient-reported symptoms [42]. Although a combination of PROs and physician-reported outcomes is the ideal standard of care when reporting RT-induced toxicity, the actual implementation remains a challenge as it is resource-intensive [43]. As the majority of the toxicity reporting is currently based on a standardized comprehensive system for reporting adverse events (e.g., CTCAE, RTOG, etc.) ([44], results obtained from our study are applicable across similar HR-PCa cases using inverse planning techniques. Secondly, DV metrics of the urethra were not available for analysis as this structure was not routinely contoured in prostate cases with conventional fractionation in our institution. While an increased in dose to the urethra has a corresponding effect on GU toxicity [45], dose prescription in the study cohort do not exceed 80 Gy and the dose distribution is homogeneous, with very small volumes of higher doses/ “hot spots” within the prostate gland. Therefore, the association of the urethra DV variables on GU toxicity in MVA should be low. Lastly, the use of DV-based metrics in MVA do not provide any geometrical information as every region of the OARs are considered equally critical, unlike the use of voxel-based metrics [46], [47]. However, the utilization of DV-based metrics as the DV variable in correlating with toxicity outcomes were commonly used, thereby enabling a robust comparison across various institutions [17], [21].

One of the main strengths of this study includes the use patient’s daily CBCTs images (5761 images) to generate D_A_ for the prostate and bladder, thus accounting for the inter-fractional organ motion that might affect the actual dose received for MVA. Although the results are not significant, the obtained single variable models can draw several important decisions to guide future dose escalation and hypofractionation strategies. For instance, like the rectum, the bladder behaves prevalently as a serial organ, thus is more sensitive to small volumes receiving high doses [48]. The dose range received by bladder V_75 Gy_ for D_A_ and D_P_ is well below our departmental planning dose constraint of ≤ 25 % [49]. Therefore, keeping within this dose limit, in addition to an acceptable bladder filling protocol and a robust image-guided RT workflow while performing dose escalation is highly recommended [11], [50]. Another point worth mentioning will be the higher D_A_ at the intermediate to high dose region (${\overset{¯}{D}}_{V30-65\;Gy}^{blad}$ and ${\overset{¯}{D}}_{ROI\;20-50\;mm}^{blad}$). Despite having a statistically significant dose differences, this result was not translated to late GU toxicity. Therefore, institutions could consider an acceptable bladder filling protocol, incorporating the use of IGRT for patients with difficulty in achieving a desired bladder filling volume. Lastly, the alternative DB-ROI method is a stronger predictor compared to the standard DV metrics in correlating with GU toxicity in the final MVA although the results were not significant. None of the DV metrics were selected during MVA. Moving forward, this study could be expanded to incorporate the use of dose surface maps (DSM) analysis with spatial information for the model-building to improve spatial dose–response correlations [51]. In addition, work is in progress to apply this model to determine the feasibility of performing dose escalation or hypofractionated regimens as well as incorporating advanced modalities, such as proton therapy to enhance the patient’s therapeutic ratio.

In conclusion, we have demonstrated that firstly, the use of DB-ROIs as surrogates for DV metrics is more predictive in MVA. Secondly, significant inter-fractional variations of the bladder occur during RT delivery as demonstrated by the higher dose received by the bladder in DV and DB-ROIs in D_A_. However, the higher bladder D_A_ observed as compared to D_P_ does not correlate to the increased risk of late GU toxicity in patients with HR-PCa. Lastly, smaller prostate volumes have a minor protective effect for Grade 2 GU toxicity. As patients were treated using inverse planned modulated techniques, the reported results serve as an excellent yardstick for toxicity predictions as compared to previously reported results using three-dimensional conformal RT. Moving forward, more research is needed in this area to enhance our knowledge pertaining to the DV-effects on late GU toxicity.

## Declaration of Competing Interest

The authors declare that they have no known competing financial interests or personal relationships that could have appeared to influence the work reported in this paper.
