# Supplementary material for: Predictors for late genitourinary toxicity in men receiving radiotherapy for high-risk prostate cancer using planned and accumulated dose
Source: Phys Imaging Radiat Oncol. 2023 Feb 2;25:100421. doi: 10.1016/j.phro.2023.100421 (PMC9932727; doi:10.1016/j.phro.2023.100421)
Supplement: Supplementary data 1 [file mmc1.docx]

**Supplemental Material**

**Predictors for late genitourinary toxicity in men receiving radiotherapy for high-risk prostate cancer using planned and accumulated dose**


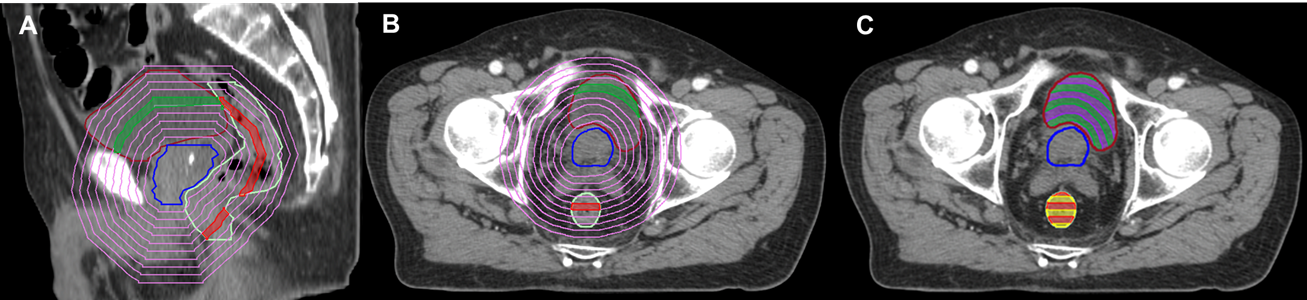


Supplemental **Fig. S1**. The region-of-interest (ROI) expanded contours (pink), starting from the prostate surface (blue), are shown overlaid on the planning CT. The rectal (red) and bladder (green) ROIs at r = 30 mm from the prostate and are shown in the sagittal (A) and axial (B) planes. (C) All ROIs for which an intersection with each OAR existed are shown in the axial plane [1].

Supplemental **Table S1**. Univariate binary logistic regression was used to analyse individual predictors with the defined late GU toxicity endpoints. A p-value of <0.05 was considered statistically significant.

| **Univariate analysis** | **Grade ≥1 late GU toxicity** | | | **Grade 2 late GU toxicity** | | |
| --- | --- | --- | --- | --- | --- | --- |
|  | Grade <1 (N=109); Grade ≥1 (N=41) | | | Grade <2 (N=139); Grade ≥2 (N=11) | | |
| **Clinical covariates** | **OR** | **95% CI** | **p-value** | **OR** | **95% CI** | **p-value** |
| Age, yrs. continuous | 0.98 | 0.921 - 1.04 | 0.43 | 0.99 | 0.90 - 1.09 | 0.84 |
| BMI, continuous | 1.02 | 0.92 - 1.12 | 0.76 | 0.86 | 0.70 - 1.07 | 0.18 |
| GS; ≤7 vs > 7 | 1.11 | 0.53 - 2.33 | 0.78 | 1.09 | 0.29 - 4.03 | 0.90 |
| T-stage; ≤ 2b vs > 2b | 1.09 | 0.53 - 2.24 | 0.82 | 1.24 | 0.36 - 4.24 | 0.74 |
| Baseline PSA, continuous | 1.00 | 1.00 - 1.01 | 0.15 | 1.00 | 0.99 - 1.01 | 0.80 |
| Anti-hypertensive; no vs yes | 0.97 | 0.47 - 1.99 | 0.93 | 0.85 | 0.25 - 2.90 | 0.79 |
| Diabetes Mellitus; no vs yes | 0.82 | 0.33 - 1.99 | 0.65 | 0.77 | 0.16 - 3.77 | 0.75 |
| Statins; no vs yes | 1.12 | 0.53 - 2.37 | 0.76 | 1.63 | 0.47 - 5.62 | 0.44 |
| TURP; no vs yes | 0.77 | 0.20 - 2.97 | 0.71 | 1.05 | 0.12 - 8.92 | 0.96 |
| ADT ≤ 6 months vs > 6 months | 1.01 | 0.96 - 1.06 | 0.75 | 1.02 | 0.94 - 1.10 | 0.69 |
| RT prescription ≤ 74 Gy vs > 74 Gy | 1.16 | 0.56 - 2.39 | 0.70 | 0.80 | 0.22 - 2.85 | 0.73 |
| **Organ volumes (cc)** |  | | | | | |
| Prostate, continuous | 0.98 | 0.96 - 1.00 | 0.07 | 0.87 | 0.79 - 0.96 | **0.01** |
| Bladder, continuous | 1.00 | 1.00 - 1.00 | 0.07 | 1.00 | 0.99 - 1.01 | 0.53 |
| **Acute toxicity** |  | | | | | |
| Grade ≥ 1; no vs yes | 0.46 | 0.20 - 1.04 | 0.06 | 0.44 | 0.12 - 1.61 | 0.22 |
| Grade ≥2; no vs yes | 0.81 | 0.35 - 1.91 | 0.64 | 0.66 | 0.14 - 3.20 | 0.61 |
| **Dose-volume (DV) covariates for D_A_** |  | | | | | |
| D_mean_ (Gy) | 1.00 | 0.94 - 1.05 | 0.92 | 0.96 | 0.87 - 1.06 | 0.38 |
| D0.03cc | 1.04 | 0.87 - 1.24 | 0.68 | 0.95 | 0.70 - 1.30 | 0.75 |
| V30 Gy | 0.99 | 0.97 - 1.02 | 0.62 | 0.99 | 0.95 - 1.03 | 0.47 |
| V35 Gy | 1.00 | 0.98 - 1.02 | 0.79 | 0.99 | 0.95 - 1.02 | 0.47 |
| V40 Gy | 1.00 | 0.98 - 1.02 | 0.91 | 0.99 | 0.95 - 1.02 | 0.44 |
| V45 Gy | 1.00 | 0.98 - 1.02 | 0.87 | 0.99 | 0.95 - 1.02 | 0.43 |
| V50 Gy | 1.00 | 0.98 - 1.03 | 0.77 | 0.98 | 0.95 - 1.02 | 0.44 |
| V55 Gy | 1.00 | 0.98 - 1.03 | 0.78 | 0.99 | 0.94 - 1.03 | 0.48 |
| V60 Gy | 1.00 | 0.98 - 1.03 | 0.87 | 0.98 | 0.94 - 1.03 | 0.47 |
| V65 Gy | 1.00 | 0.98 - 1.03 | 0.88 | 0.98 | 0.92 - 1.03 | 0.43 |
| V70 Gy | 1.00 | 0.98 - 1.04 | 0.92 | 0.97 | 0.90 - 1.04 | 0.38 |
| V75 Gy | 0.99 | 0.98 - 1.05 | 0.74 | 0.94 | 0.84 - 1.06 | 0.32 |
| **DB-ROI for D_A_ (Gy)** |  | | | | | |
| D- 5 mm | 1.09 | 0.92 - 1.28 | 0.32 | 1.01 | 0.77 - 1.33 | 0.95 |
| D- 10 mm | 1.05 | 0.93 - 1.18 | 0.43 | 1.03 | 0.84 - 1.25 | 0.80 |
| D- 15 mm | 1.00 | 0.93 - 1.08 | 0.99 | 1.01 | 0.89 - 1.15 | 0.86 |
| D- 20 mm | 0.98 | 0.92 - 1.04 | 0.53 | 1.01 | 0.91 - 1.13 | 0.86 |
| D- 25 mm | 0.96 | 0.90 - 1.02 | 0.19 | 0.98 | 0.88 - 1.09 | 0.67 |
| D- 30 mm | 0.95 | 0.89 - 1.01 | 0.10 | 0.95 | 0.86 - 1.05 | 0.35 |
| D- 35 mm | 0.95 | 0.89 - 1.01 | 0.08 | 0.94 | 0.85 - 1.04 | 0.24 |
| D- 40 mm | 0.96 | 0.91 - 1.01 | 0.14 | 0.96 | 0.89 - 1.04 | 0.36 |
| D- 45 mm | 0.96 | 0.91 – 0.99 | 0.07 | 0.98 | 0.91 - 1.05 | 0.53 |
| D- 50 mm | 0.96 | 0.93 – 0.99 | **0.04** | 0.99 | 0.93 - 1.06 | 0.86 |
| **DV covariates for D_P_** |  | | | | | |
| D_mean_ (Gy) | 0.99 | 0.94 - 1.05 | 0.80 | 0.96 | 0.86 - 1.07 | 0.43 |
| D0.03cc | 1.05 | 0.88 - 1.24 | 0.61 | 0.96 | 0.71 - 1.29 | 0.77 |
| V30 Gy | 1.00 | 0.97 - 1.02 | 0.70 | 0.99 | 0.95 - 1.03 | 0.73 |
| V35 Gy | 0.99 | 0.97 1.02 | 0.59 | 0.99 | 0.95 - 1.03 | 0.50 |
| V40 Gy | 1.00 | 0.97 - 1.02 | 0.89 | 0.99 | 0.94 - 1.03 | 0.51 |
| V45 Gy | 1.00 | 0.97 - 1.02 | 0.88 | 0.98 | 0.94 - 1.03 | 0.44 |
| V50 Gy | 1.00 | 0.98 - 1.03 | 0.96 | 0.98 | 0.94 - 1.03 | 0.42 |
| V55 Gy | 1.00 | 0.98 - 1.03 | 0.90 | 0.98 | 0.93 - 1.03 | 0.44 |
| V60 Gy | 1.00 | 0.97 - 1.03 | 0.86 | 0.98 | 0.93 - 1.04 | 0.47 |
| V65 Gy | 1.01 | 0.97 - 1.04 | 0.76 | 0.98 | 0.92 - 1.04 | 0.50 |
| V70 Gy | 1.01 | 0.97 - 1.05 | 0.66 | 0.98 | 0.90 - 1.05 | 0.53 |
| V75 Gy | 1.01 | 0.95 - 1.07 | 0.73 | 0.97 | 0.87 - 1.08 | 0.58 |
| **DB-ROI for D_P_ (Gy)** |  | | | | | |
| D- 5 mm | 1.08 | 0.89 - 1.30 | 0.44 | 0.97 | 0.71 - 1.35 | 0.87 |
| D- 10 mm | 1.06 | 0.92 - 1.23 | 0.44 | 1.02 | 0.80 - 1.31 | 0.87 |
| D- 15 mm | 0.99 | 0.92 - 1.07 | 0.82 | 1.02 | 0.89 - 1.18 | 0.76 |
| D- 20 mm | 0.97 | 0.91 - 1.04 | 0.34 | 1.01 | 0.90 - 1.13 | 0.87 |
| D- 25 mm | 0.95 | 0.89 - 1.02 | 0.14 | 0.96 | 0.86 - 1.07 | 0.49 |
| D- 30 mm | 0.95 | 0.89 - 1.01 | 0.08 | 0.95 | 0.85 - 1.05 | 0.27 |
| D- 35 mm | 0.94 | 0.89 - 1.01 | 0.07 | 0.94 | 0.85 - 1.04 | 0.24 |
| D- 40 mm | 0.96 | 0.91 - 1.01 | 0.13 | 0.97 | 0.90 - 1.06 | 0.51 |
| D- 45 mm | 0.96 | 0.91 - 1.01 | 0.14 | 0.98 | 0.90 - 1.06 | 0.63 |
| D- 50 mm | 0.96 | 0.93 – o.99 | **0.04** | 1.01 | 0.94 - 1.08 | 0.89 |

Abbreviations: OR = odds ratio, CI = confidence interval, D_P_ = Planned dose, D_A_ = Accumulated dose, BMI = body mass index, GS = Gleason score, BL PSA = baseline prostate specific antigen, TURP = transurethral resection of the prostate, ADT = androgen deprivation therapy, DB-ROI = dose-based region of interest

Reference

[1] Ong A, Knight K, Panettieri V, Dimmock M, Tuan JKL, Tan HQ, et al. Application of an automated dose accumulation workflow in high-risk prostate cancer - validation and dose-volume analysis between planned and delivered dose. Med Dosim. 2022;47:92-7.
